# Toll/IL-1 receptor domain-containing adaptor protein plays a crucial role in macrophage-mediated hepatic stellate cell activation in alcoholic liver disease

**DOI:** 10.3389/fcell.2026.1883301

**Published:** 2026-08-04

**Authors:** Pramod Patidar, Alexander G. Obukhov, Sheikh A. Tasduq, Luciano Saso, Mirza S. Baig

**Affiliations:** 1 Mehta Family School of Biosciences and Biomedical Engineering, Indian Institute of Technology Indore (IITI), Indore, India; 2 Department of Anatomy, Cell Biology & Physiology, Indiana University School of Medicine, Indianapolis, IN, United States; 3 Stark Neurosciences Research Institute, Indiana University School of Medicine, Indianapolis, IN, United States; 4 PK-PD and Toxicology Division, Council for Scientific and Industrial Research, Indian Institute of Integrative Medicine, Indore, India; 5 Institute of Evolutionary Medicine, University of Zurich, Zurich, Switzerland; 6 Department of Physiology and Pharmacology “Vittorio Erspamer”, Sapienza University, Rome, Italy

**Keywords:** alcoholic liver fibrosis, hepatic inflammation, hepatic stellate cell, macrophage, TIRAP silencing

## Abstract

**Background:**

Alcoholic liver disease (ALD) remains a significant global health issue, marked by chronic liver injury and progressive accumulation of extracellular matrix (ECM), ultimately leading to fibrosis. A key driver of this fibrotic process is the activation of hepatic stellate cells (HSCs), largely influenced by inflammatory signals from immune cells—particularly macrophages. This study explores the specific signalling mechanisms in macrophages that contribute to HSC activation in the context of alcohol exposure, aiming to identify new therapeutic targets.

**Methods:**

To mimic the disease condition, THP-1-derived macrophages were stimulated with ethanol and lipopolysaccharide (LPS). Cytokine expression was quantified by RT-qPCR, while the activation of TIRAP, MAPKs, and fibrotic markers in THP-1-derived macrophages and LX-2 cells was evaluated by western blotting and immunofluorescence staining.

**Result:**

Activation was indicated by increased expression of pro-inflammatory and fibrogenic factors, including IL-1β, TNF-α, IL-6, TGF-β, and PDGF-α. Signalling analysis revealed heightened phosphorylation of TIRAP (Toll/IL-1 receptor domain-containing adaptor protein), along with activation of downstream MAPK pathways (p38, ERK, JNK) and NF-κB. The conditioned media from these activated macrophages was applied to HSC cultures, resulting in elevated levels of fibrotic markers such as α-smooth muscle actin (α-SMA) and collagen, confirming HSC activation. Importantly, silencing TIRAP in macrophages significantly reduced the expression of these markers in HSCs, suggesting a key role for TIRAP in driving fibrogenesis.

**Conclusion:**

Overall, these findings underscore the importance of TIRAP- mediated signalling in macrophages as a central mechanism in alcohol- induced liver fibrosis, and propose macrophage TIRAP as a promising target for therapeutic intervention in ALD.

## Graphical Abstract

**Figure:**
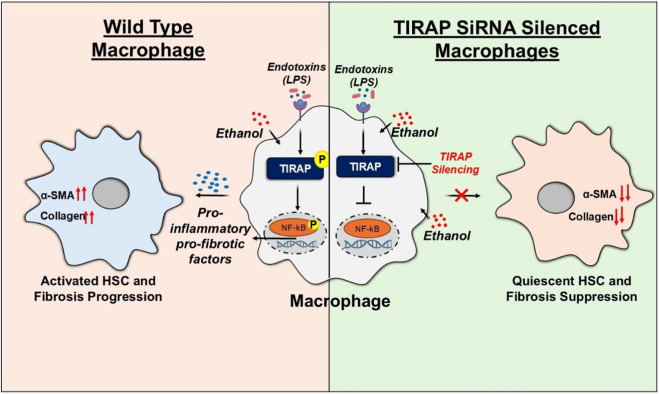
Representation of macrophage specific TIRAP mediated hepatic stellate cell activation during alcoholic liver fibrosis.

## 1 Introduction

Liver diseases claim the lives of over two million individuals each year worldwide, of which 1 million were diagnosed with cirrhosis, a severe liver fibrosis (Ginès et al., 2021). Liver fibrosis is a common pathological condition associated with inflammatory and chronic liver diseases. Prolonged and persistent inflammation is an important contributor to the progression of liver fibrosis (Zhang et al., 2016). It is a complex and multistage process orchestrated by the interaction of various parenchymal and non-parenchymal cells, such as Kupffer cells, liver endothelial cells, hepatic stellate cells, and hepatocytes (Acharya et al., 2021). In the healthy liver, hepatic stellate cells are the major storage sites for Vitamin A and the production house of growth factors important for hepatocyte regeneration. However, overactivated hepatic stellate cells also secrete increased amounts of various cytokines, growth factors, chemokines, extracellular matrix (ECM) proteins, and adhesion molecules that may facilitate liver fibrosis (Acharya et al., 2021). The overactivation begins with liver injury to the development of fibrosis, which eventually leads to cirrhosis. Simultaneously this event can be regulated by the activation of macrophages too (Tan et al., 2024). Therefore, studying the interaction between liver macrophage-like Kupffer cells and hepatic stellate cells has been of great importance for the development of therapeutic strategies for liver fibrosis and cirrhosis.

The HSCs that remain in a quiescent stage, exhibit controlled expression of α-SMA, collagen, and other extracellular proteins. However, during chronic liver inflammation, HSCs upregulate those expression and undergo myofibroblast-like cell transformation (Zhang et al., 2022). Such transformation of HSCs into myofibroblast like cells are induced by various bacterial and viral pathogenic factors, injury, cytokines, and growth factors. Additionally, this phenomenon is closely linked to macrophages which serve as crucial driver of HSCs activation (Widowati et al., 2026).

Liver-resident Kupffer cells and peripheral macrophages play a significant role in maintaining immune homeostasis in the liver; however, when overactivated, they can lead to liver injury and subsequent liver fibrosis (Suraweera, 2015) It is well established that during alcoholic liver diseases (ALD), due to the leaky gut, serum endotoxins levels, primarily LPS, are significantly elevated (Fang et al., 2004). LPS-activated liver-resident macrophages produce increased amounts of various proinflammatory cytokines and growth factors via the TLR4 signalling pathway (Fang et al., 2004). The prolonged and imbalanced expression of the cytokines and growth factors causes chronic hepatic inflammation and activation of hepatic stellate cells.

Toll/interleukin-1 receptor domain-containing adapter protein or MyD88 adapter-like protein (TIRAP/MAL) act as a bridge during TLRs signaling (Atre et al., 2023). It is the most upstream adaptor protein in inflammatory signalling pathways, which facilitates the activation of nuclear factor kB (NF-kB) and activated protein-1 (AP-1) transcription factors, ultimately leading to the expression of various inflammatory cytokines (Liu et al., 2008). TIRAP has also been shown to involve in the variety of protein-protein interaction in various inflammatory conditions (Rajpoot et al., 2021). Recent studies have reported the essential role of TLR4-mediated signaling pathways in ethanol-induced inflammation (Holloway et al., 2023). Though, TIRAP involvement in inflammatory conditions is known, but its precise role in the pathogenesis of alcoholic liver fibrosis is still under investigation. To address this critical gap in knowledge, our study aims to dissect the specific, TIRAP-mediated paracrine axis between macrophages and HSCs under alcoholic conditions. By focusing on the intercellular crosstalk, this study reveals a potential mechanistic pathway. Ultimately, our work highlights macrophage TIRAP as a precise, novel therapeutic target to halt the progression of alcoholic liver fibrosis.

## 2 Methods and materials

### 2.1 Cell culture

The THP-1 cell line, originally isolated from the peripheral blood of an acute monocytic leukemia patient were sourced from NCCS Pune. The LX-2 cell line, derived from primary human hepatic stellate cells was a gift from Dr. Sheikh Tasduq Abdullah (CSIR–Indian Institute of Integrative Medicine, Jammu). These cell lines were maintained in Roswell Park Memorial Institute 1640 (RPMI) and Dulbecco’s Modified Eagle Medium (DMEM), respectively, purchased from Gibco Thermo Fisher Scientific India, and supplemented with 10% FBS (Gibco, Thermo Fisher). The culture medium was further supplemented with 1% penicillin/streptomycin. Cells were grown in an incubator at 37 °C with 5% CO2.

### 2.2 Macrophage conditioned medium preparation and treatment

The THP-1 monocytes differentiated into macrophages using 25 ng/mL phorbol 12-myristate 13-acetate (PMA; Sigma-Aldrich) in the culture medium for 16 hrs. Followed by PMA treatment culture medium was replaced with fresh media and cells subjected to treatment with LPS (500 ng/mL), ethanol (40 mM), and a combination of LPS and ethanol (LE) for 24 h. Following the treatment period, the culture medium was aspirated and replaced with fresh medium. Subsequently, the conditioned medium (CM) was collected after 24 h, centrifuged, and filtered using a 0.2 µm filter to eliminate cellular debris. This CM was utilised for the treatment of the LX-2 cell line to determine the effect of macrophages on LX-2 activation.

### 2.3 siRNA transfection and TIRAP silencing in macrophages

THP-1 macrophages were seeded in 6-well plates at a density of 0.5 × 10^5^ cells per well, and upon reaching 80% confluency, the cells were treated with PMA to induce differentiation. Following this, cells were transfected with TIRAP-specific siRNA at concentrations of 10 nM, 25 nM, and 50 nM. For transfection, TIRAP siRNA was diluted in 100 µL of serum-free medium, and separately, 5 µL of Lipofectamine transfection reagent was diluted in 100 µL of serum-free medium. Both solutions were combined and incubated at room temperature for 45 min to allow complex formation. The resulting siRNA-lipid complexes were then added to macrophages in wells containing 800 µL of serum-free medium, and the cells were incubated with the complexes for 4–6 h. Following this, 1 mL of complete medium supplemented with 2× serum concentration was added to each well, and incubation continued for an additional 24 h. Silencing efficiency was confirmed by Western blot and confocal microscopy analyses. The same transfection protocol was used to generate conditioned media; thereafter, cells were treated with LPS, ethanol, and LE. While a well-validated commercial siRNA targeting TIRAP was utilized to ensure target specificity, a scrambled siRNA control was not included in the present protocol.

### 2.4 Quantitative real-time polymerase chain reaction (qPCR)

A qRT-PCR analysis was performed to investigate the cytokine expression in THP-1 cells, using the standardised procedures described earlier. Following the treatment, cell pellets were collected, and RNA isolation was carried out using TRIZol reagent (Takara, Shiga, Japan). Subsequently, cDNA synthesis was conducted using PrimeScript RT Master Mix (Takara, Shiga, Japan). The expression levels of cytokines in THP-1 cells and fibrotic markers in LX-2 cells were quantified via qRT-PCR with SYBR green (Applied Biosystems, Waltham, United States) and gene-specific primers (Supplementary Table S1) on Stepone (Thermo Fisher Scientific). The gene expression level was normalised to that of GAPDH.

### 2.5 Cellular immunofluorescence

THP-1 and LX-2 cells were seeded on coverslips in 12-well plates (0.5 × 10^5^ cells/well). For fluorescence staining, after treatment, cells were washed with PBS and fixed in 4% paraformaldehyde for 15 min and permeabilized with 0.1% Triton-X 100 for 10 min. Cells were then blocked with 5% blocking reagent at room temperature to reduce nonspecific binding. Then, cells were incubated overnight at 4 °C with primary antibodies (dilution 1:250) against phospho-TIRAP, c-JUN, Phospho-NF-kB, Collagen, and α-SMA (Supplementary Table S2). Followed by incubation with FITC conjugated goat anti-mouse Alexa Fluor 488, chicken anti-rabbit Alexa Fluor 594 and chicken anti goat Alexa 594 secondary antibodies (Supplementary Table S2). Nuclear counterstaining was carried out using a 1 mg/mL DAPI solution in PBS (Thermo Fisher Scientific, Cat. No. 62248, Thermo Fisher Scientific, Germany) according to the manufacturer’s instructions. Stained cells were analysed using an Olympus confocal laser scanning microscope.

### 2.6 Western blot analysis

THP-1 and LX-2 cells were seeded in 6-well plates (1 × 10^5^ cells/well). For Western blot analysis, after treatment, cells were washed with chilled PBS and lysed at 4 °C in RIPA lysis buffer containing protease inhibitors. After 2 h, the cell lysate was centrifuged at 13,000 RPM for 30 min at 4 °C, and the supernatant was collected and used as a protein sample for further experiments. Protein samples were resolved in a 12% SDS gel, followed by an overnight transfer of protein to a nitrocellulose membrane. After the transfer membrane was blocked with 5% BSA for 1 h, followed by TBST 3X washing at pH 7.6. The membrane was incubated overnight with primary antibody (Supplementary Table S2) at a dilution of 1:1000 in TBST Buffer. After washing, the membrane was incubated with secondary antibody (dilution 1:5000) for 2 h at RT. For quantitative analysis of blots, HRP substrate was used, and detection was done by Vilber Fusion Solo X image reader. Further band intensity was quantified using ImageJ.

### 2.7 Statistical analysis

The experimental results were obtained from three independent biological replicates. A one-way ANOVA followed by Tukey’s multiple comparison post-hoc test was implemented in the statistical evaluation of the data. P values were estimated using GraphPad Prism version 8, and p values of 0.01, 0.001, and 0.0001 were considered statistically significant and represented by *, **, and ***, denoting upregulation/downregulation, respectively.

## 3 Results

### 3.1 Alcohol regulates TIRAP expression and activation in macrophages

**FIGURE 1:**
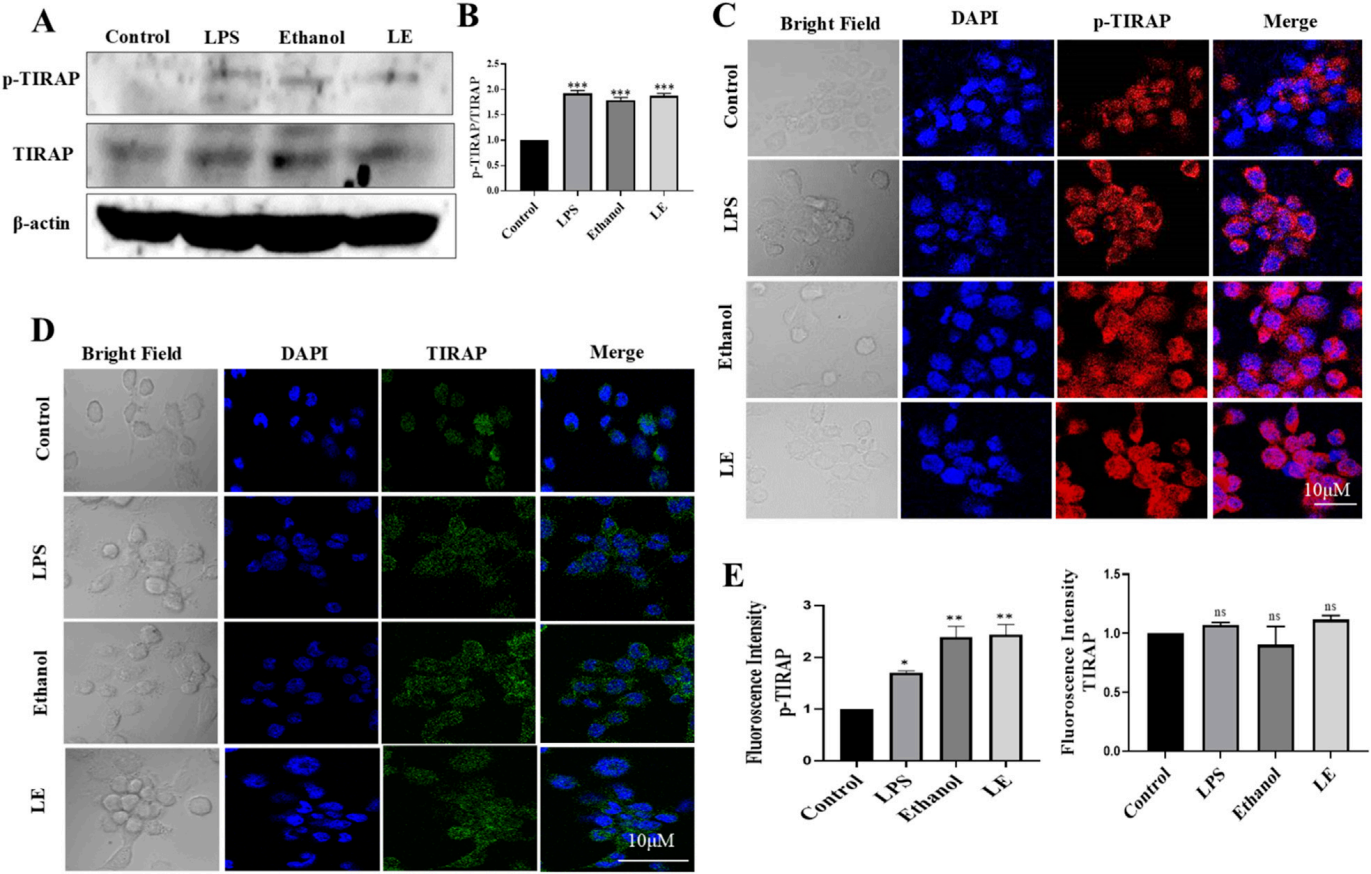
Ethanol treatment promotes TIRAP activation in THP-1 macrophages. **(A,B)** THP-1 macrophage exposed to ethanol (40 Mm), LPS (1 μ μ g/mL), and LE for 24 h and TIRAP phosphorylation was analysed by western blotting using the indicative antibodies and band intensity was quantified by imageJ software. **(C)** Confocal microscopy images showing the cellular phosphorylation of TIRAP by immunostaining with their specific primary antibodies for phosphor-TIRAP. The secondary antibodies were Alexa Fluor 594-tagged (red) for phosphor-TIRAP along with their quantification by imageJ software. **(D)** Confocal microscopy images showing the cellular expression of TIRAP by immunostaining with their specific primary antibodies for TIRAP. The secondary antibodies were Alexa Fluor 488-tagged (green) for TIRAP. **(E)** represents the quantification of fluorescence intensity of phospho-TIRAP and TIRAP by imageJ software. All data are representative of three independent experiments and are presented as the mean ± SD, (One-way Anova test). ****P ≤ 0.00001; ***P ≤ 0.0001; **P ≤ 0.001; *P ≤ 0.01.

During alcoholic conditions to elucidate the role of TIRAP in the crosstalk of macrophage and HSCs, we first investigated the effect of ethanol on monocyte-derived THP-1 macrophages. Differentiated THP-1 cells were exposed to ethanol, LPS, and a mixture of ethanol and LPS (LE) for 24 h, and expression and activation/phosphorylation of TIRAP were assayed. As shown in Figure 1A, exposure to ethanol, LPS, and LE significantly increases TIRAP phosphorylation in THP-1 macrophages (Figure 1A, Supplementary Figure S1C). This increase in phosphorylation or TIRAP activation was further confirmed by immunofluorescence analysis (Figures 1C,E). However, at the expression level, there was no change in TIRAP expression (Figures 1D,E). Taken together, these results indicate that in THP-1 macrophages, ethanol regulates TIRAP activation.

### 3.2 NF-κB activation in alcohol-stimulated macrophages

**FIGURE 2:**
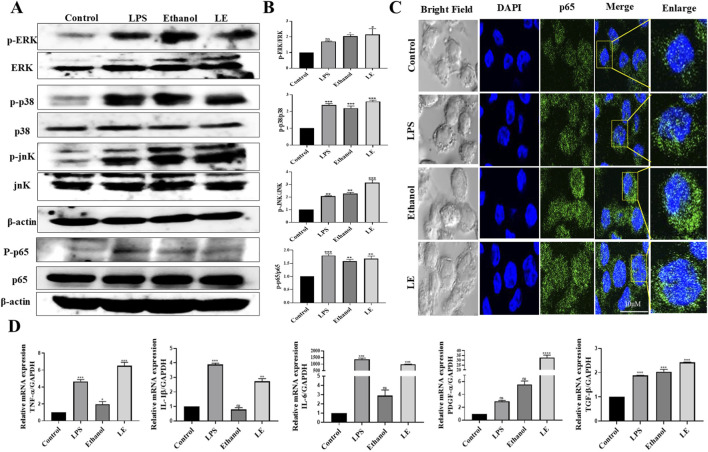
Ethanol treatment promotes NF-kB phosphorylation in THP-1 macrophages. **(A,B)** THP-1 macrophages were stimulated with LPS (1 μ μ g/mL), ethanol (40 mM) and LE for 24 h. Immunoblot analysis for MAPKs revealed a significant upregulation of p38, JNK, ERK and transcription factor NF-kB phosphorylation along with quantification of band intensity by ImageJ software. **(C)** Confocal microscopy images showing the nuclear translocation of p65 by immunostaining with their specific primary antibodies for p65. The secondary antibodies were Alexa Fluor 488-tagged (Green) for p65. **(D)** Relative mRNA expression of pro-inflammatory cytokines (, IL-1β, IL-6, TNFα), growth factor (TGF-β, PDGF), was determined after normalizing to GAPDH’s mRNA expression levels. All data are representative of three independent experiments and are presented as the mean ± SD, (One-way Anova test). ***P ≤ 0.0001; **P ≤ 0.001; *P ≤ 0.01.

TIRAP is the most upstream key adaptor in Toll-like receptor (TLR) signalling, particularly for TLR2 and TLR4. It facilitates the activation of downstream signalling pathways, which include activation of certain transcription factors such as NF-κB. Based on this, we examined the effect of ethanol on TIRAP-dependent activation of MAP Kinases. We found that treatment with ethanol, LPS, or LE for 24 h led to a significant activation of MAPKs, including p38, JNK, and ERK, in THP-1 macrophages, compared to the control THP-1 macrophages (Figure 2A). The phosphorylation of MAPKs was greater in THP-1 macrophages treated with ethanol and LPS, compared to control THP-1 macrophages (Figures 2A,B). These MAP kinases can activate various downstream transcription factors. Therefore, we further explored the effect of ethanol on NF-κB activation in THP-1 macrophages. We found that ethanol induced substantial activation of the NF-κB transcription factor in THP-1 macrophages (Figure 2A, Supplementary Figure S1C). NF-κB activation was further confirmed by its nuclear translocation using immunofluorescence analysis (Figure 2C). These results indicate that ethanol can modulate NF-κB activation via a TIRAP-dependent pathway.

### 3.3 Alcohol induces the expression of inflammatory mediators in macrophages

We next examined the expression of cytokines in THP-1 macrophages treated with ethanol, LPS, and a combination of LPS and ethanol. Our finding indicates that proinflammatory cytokines, including IL-1β, IL-6 and TNF-α, were significantly upregulated in all conditions compared to the control, except IL-1β in ethanol-treated macrophages (Figure 2D). Further, we also determined the expression levels of growth factor and profibrotic molecule TGF-β and PDGF-α. We found that ethanol significantly upregulated the expression of TGF-β and PDGF-α (Figure 2D). These findings suggest that the presence of ethanol in the system induces the expression of both pro-inflammatory and anti-inflammatory cytokines, growth factors, and profibrotic molecules. This ensures that under alcoholic conditions, macrophages may exhibit both pro and anti-inflammatory phenotypes and influence disease progression.

### 3.4 Alcohol-stimulated macrophages induce profibrotic response in HSCs

**FIGURE 3:**
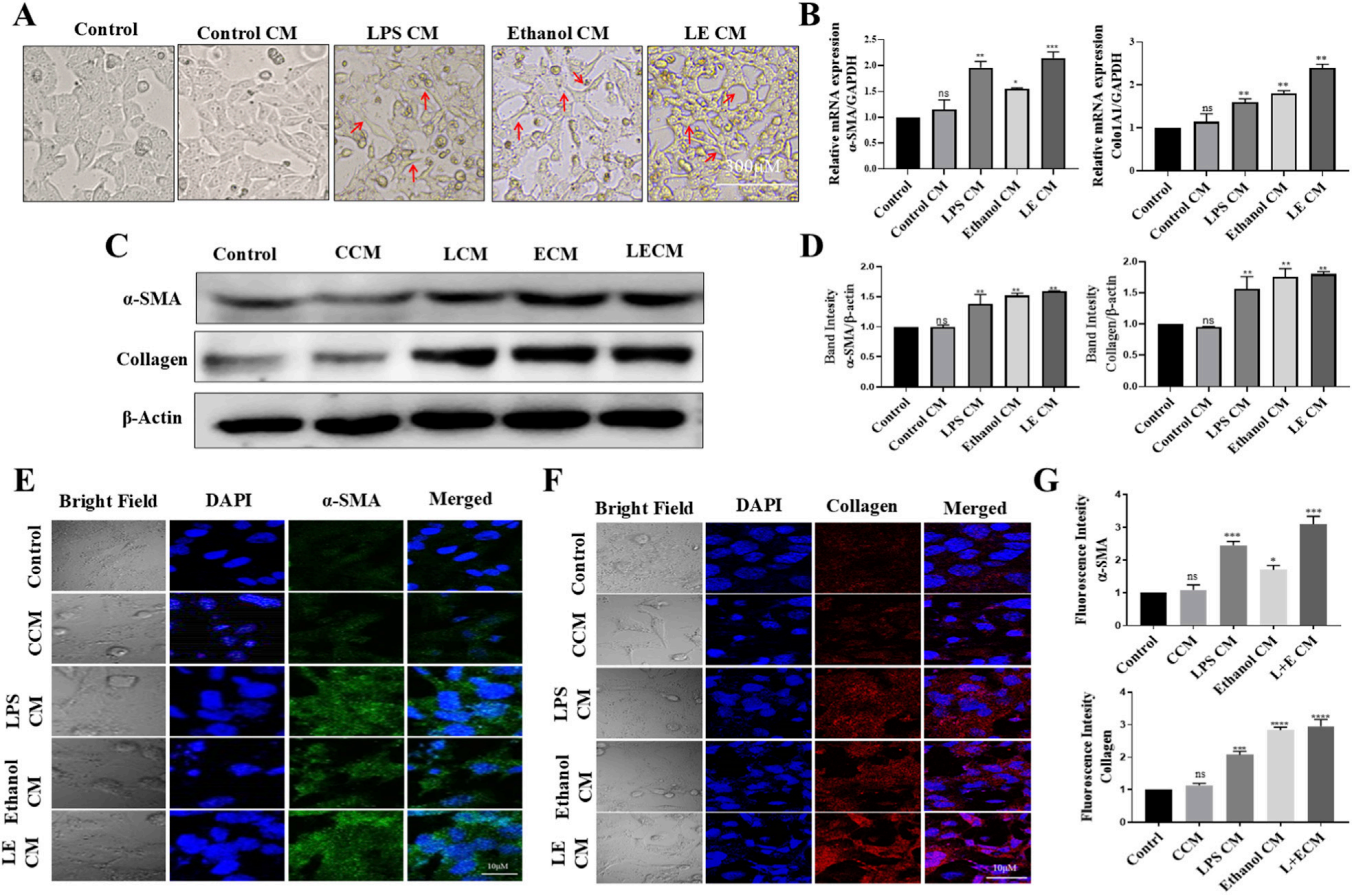
Macrophage-mediated HSCs activation. THP-1 macrophages exposed to LPS, ethanol and LE and generated condition media was use for treatment of HSCs for 24 h s. **(A)** HSCs treated with THP-1 macrophage-conditioned media exhibit distinct morphological changes compared to controls, while HSCs exposed to LPS, ethanol, and LE-conditioned media display prominent star-shaped and spiky morphology, as highlighted by the red arrow. **(B)** Total RNA was isolated from HSCs treated with macrophage-conditioned media, and relative mRNA expression of α-SMA and collagen genes was evaluated by qRT-PCR and normalized for the expression of GAPDH. **(C,D)** Immunoblot analysis showed increased expression of α-SMA and collagen in HSCs treated with condition media derived from THP-1 macrophage and their quantification of band intensity by image J software. **(E–G)** Confocal microscopy images showing the cellular expression of α-SMA and collagen by immunostaining with their specific primary antibodies for α-SMA and collagen. The secondary antibodies were Alexa Fluor 488-tagged (green) for α-SMA and Alexa Fluor 488-tagged (red)for collagen along with their quantification of fluorescence intensity by imageJ software. All data are representative of three independent experiments and are presented as the mean ± SD, (One-way Anova test). ***P ≤ 0.0001; **P ≤ 0.001; *P ≤ 0.01.

Next, we sought to determine whether alcohol itself or macrophage-derived profibrotic factors modulate the HSC state. To identify the involvement of TIRAP-mediated signalling in the crosstalk between macrophages and HSCs, we investigate the effect of CM derived from THP-1 macrophages following LPS, ethanol, and LE treatments, on HSCs activation. We found that HSCs treated with CM show activated morphology. Figure 3A shows that HSCs treated with CM exhibit spike formation and assume a star-like shape (marked by red arrows) as compared to the control conditions. To further confirm CM-mediated activation of HSCs, we assessed the expression of specific biomarkers, including collagen and smooth muscle actin (α-SMA), in HSCs. mRNA expression of α-SMA and collagen was upregulated in HSCs treated with conditioned media compared with control conditions (Figure 3B). Consistently, we observed upregulated protein levels of α-SMA and collagen by Western blotting (Figures 3C,D) and immunofluorescence analysis (Figures 3E–G). These findings suggest that under alcoholic conditions, TIRAP-mediated activation of THP-1 macrophages may promote subsequent HSC activation.

### 3.5 TIRAP silencing in macrophages

**FIGURE 4:**
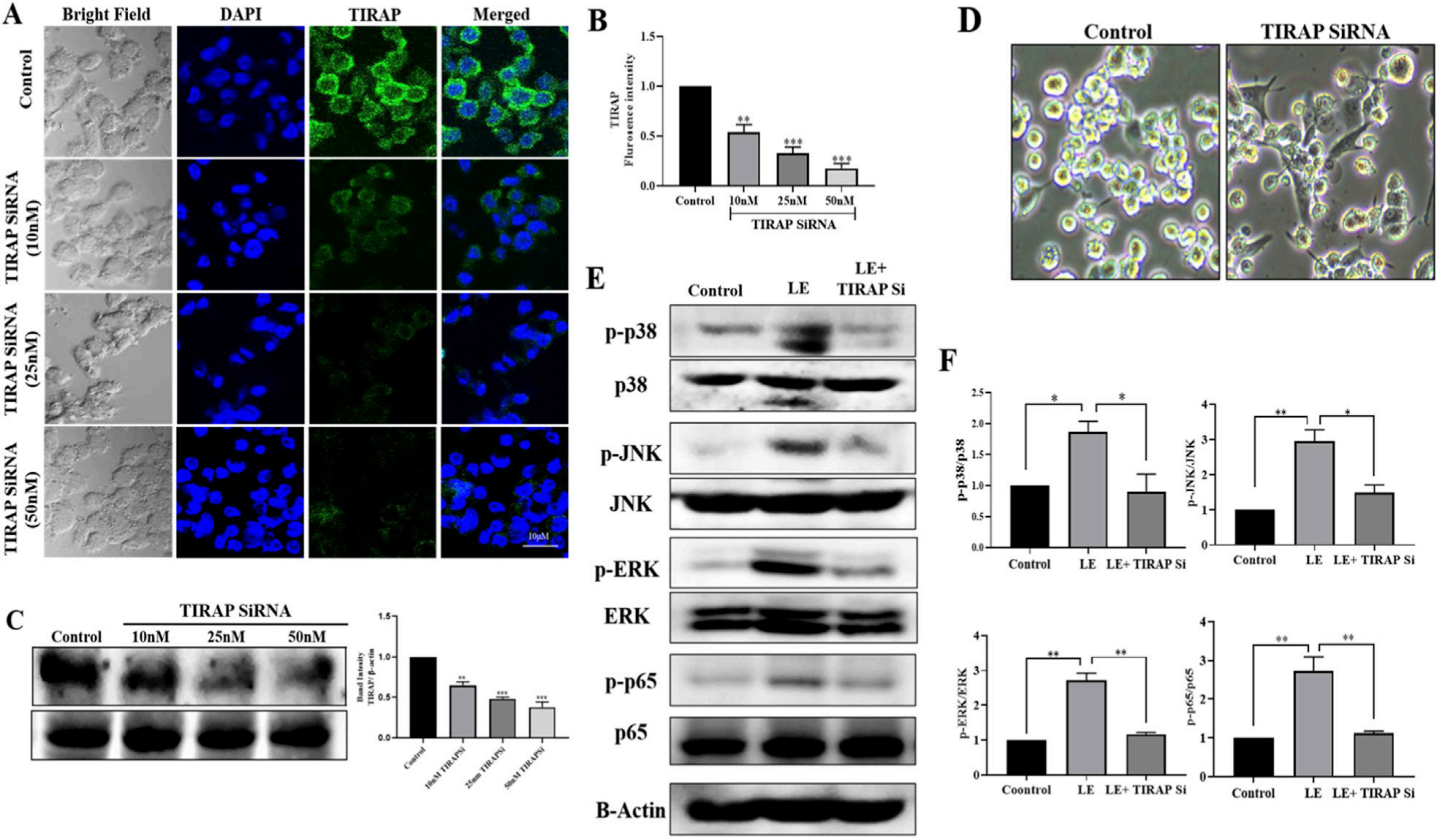
TIRAP silencing in THP-1 macrophages. **(A,B)** Confocal microscopy images showing the degradation of TIRAP in THP-1 macrophage treated with different concentrations of TIRAP SiRNA 10 nM, 25 nM, and 50 nM for 24 h, the cellular expression of TIRAP by immunostaining with their specific primary antibodies for TIRAP. The secondary antibodies were Alexa Fluor 488-tagged (green) for TIRAP. **(C)** Immunoblot of TIRAP expression in THP-1 macrophages and band intensity quantification by imageJ. **(D)** THP-1 macrophages morphology treated with 25 nM TIRAP siRNA for 24 h **(E,F)** Immunoblot analysis for MAPKs in THP-1 macrophage treated with TIRAP siRNA 25 nM concentration, revealed a significant downregulation-regulation of p38, JNK, ERK and transcription factor NF-kB phosphorylation along with quantification of band intensity by ImageJ software All data are representative of three independent experiments and are presented as the mean ± SD, (One-way Anova test). ***P ≤ 0.0001; **P ≤ 0.001; *P ≤ 0.01.

To validate the role of macrophage-specific TIRAP signalling in macrophage-HSCs crosstalk, TIRAP expression was knocked down in THP-1 macrophages prior to generating the CM. TIRAP knockdown was confirmed by confocal microscopy (Figures 4A,B) and western blot analysis (Figure 4C). Results showed that a 25 nM concentration of TIRAP-specific siRNA was efficient in knocking down the TIRAP expression in macrophages. We also found that TIRAP-knockdown macrophages exhibited an elongated morphology compared with controls (Figure 4D). Furthermore, our findings indicated that, TIRAP silencing in macrophages also reduced the activation of MAPKs including p38, ERK, and JNK, resulting downregulation of NF-kB activation (Figures 4E,F). This result confirms the TIRAP knockdown in macrophages.

### 3.6 Macrophage-targeted TIRAP knockdown suppresses activation of HSCs

**FIGURE 5:**
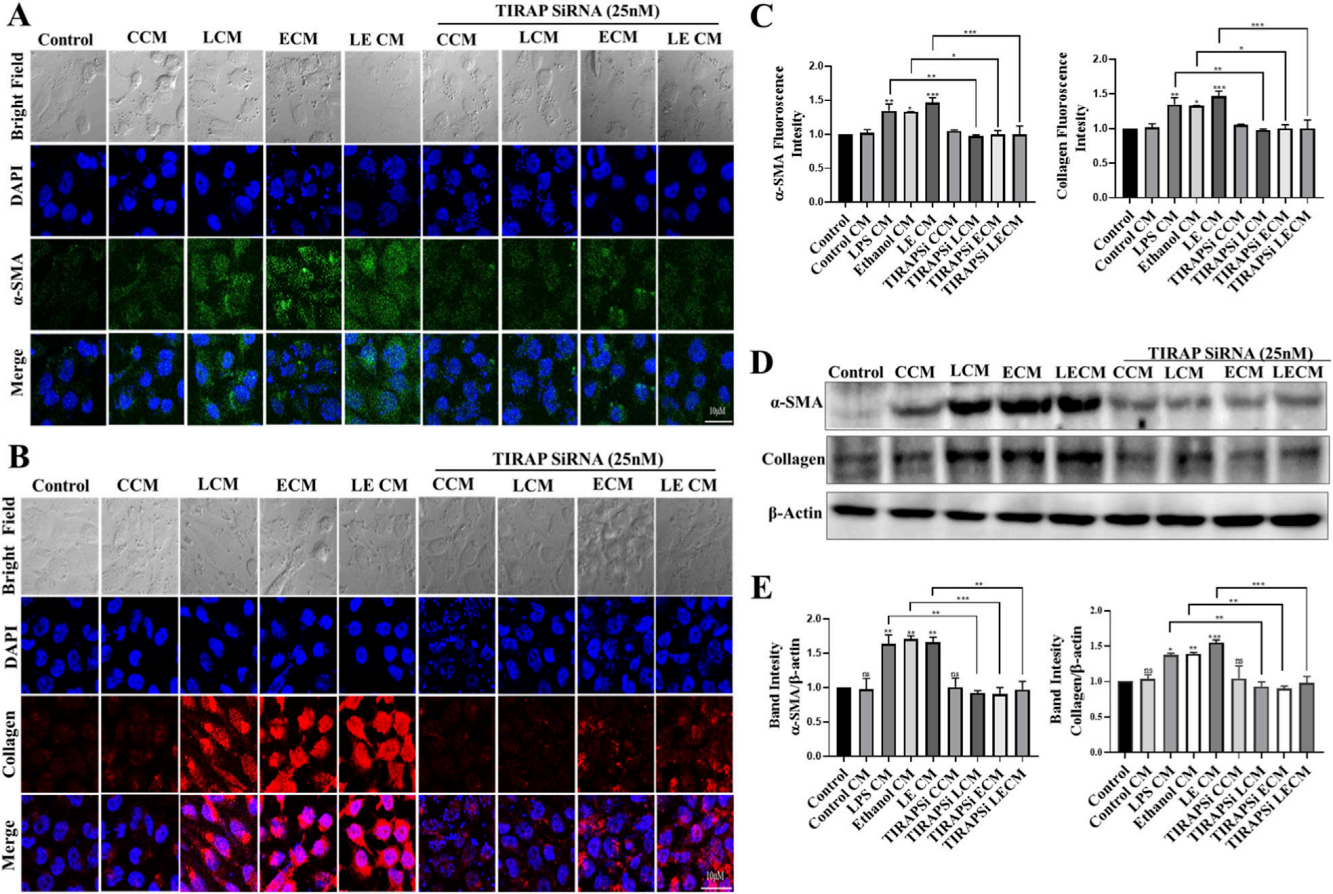
Effect of macrophage specific-TIRAP knocked out macrophage condition media on HSCs activation. Wild type and TIRAP knocked out THP-1 macrophages exposed to LPS, ethanol and LE and generated condition media was used for treatment of HSCs for 24 h s. **(A–C)** Confocal microscopy images showing the cellular expression of α-SMA and collagen by immunostaining with their specific primary antibodies for SMA and collagen. The secondary antibodies were Alexa Fluor 488-tagged (green) for α-SMA and Alexa Fluor 488-tagged (red)for collagen along with their quantification of fluorescence intensity by ImageJ software. **(D,E)** Immunoblot analysis showed cellular protein expression of α-SMA and collagen in HSCs treated with condition media derived from Wild type and TIRAP knocked out THP-1 macrophage and their quantification of band intensity by image J software. All data are representative of three independent experiments and are presented as the mean ± SD, (One-way Anova test). ***P ≤ 0.0001; **P ≤ 0.001; *P ≤ 0.01.

Subsequently, to substantiate the importance of macrophage-specific TIRAP in HSC activation, we generated the CMs from both wild-type and TIRAP-knockdown macrophages, which were used to stimulate HSC activation. We found that the HSCs treated with CM derived from TIRAP-knockdown macrophages exhibited no activation or morphological changes compared to HSCs treated with wild-type macrophage-CM (Supplementary Figure S2). Similar results were obtained at the molecular level when we explored the effect of CM from TIRAP-knockdown macrophages and wild-type macrophages. In HSCs treated with TIRAP-knockdown macrophages, CM showed significantly reduced expression of collagen and α-SMA compared to HSCs treated with wild-type CM, confirmed by immunofluorescence analysis (Figures 5A–C). Further, decreased expression of collagen and α-SMA was found at the protein level by Western blot analysis (Figures 5D,E). These findings signify that TIRAP is a crucial factor in macrophage-HSCs crosstalk. Because silencing of TIRAP in macrophages potentially abrogated their capacity to induce HSC activation, highlighting the activation of TIRAP-mediated signalling in macrophages induces a profibrotic microenvironment.

## 4 Discussion

Liver fibrosis is the end stage of most chronic liver diseases. Liver fibrosis and hepatic stellate cell activation may result from bacterial and viral infections, and chronic alcohol consumption (Poynard et al., 2003). It is a progressive and multistage process which involves the interaction of various parenchymal and non-parenchymal cells, such as tissue resident and circulating macrophage, liver endothelial cells, hepatocytes and hepatic stellate cells (Berumen et al., 2021). Various studies have suggested that macrophages play a critical role in the development and progression of chronic liver diseases (Ju and Tacke, 2016). It was suggested that under alcoholic condition, macrophages have the capacity to activate HSCs via secretion of profibrotic factors, thereby contributing to the progression of liver fibrosis (Pastore et al., 2022). Chronic alcohol consumption impairs the function of the gut barrier and increases gut permeability, leading to an imbalance in gut microbiota populations. This facilitates increased blood serum endotoxin (LPS) level (Parlesak et al., 2000). Endotoxin LPS is a potent activator of the TLR4 receptor which is widely expressed throughout the liver, including in both parenchymal and non-parenchymal cells (Park and Lee, 2013). Activation of TLR4 initiates a series of signalling pathways that ultimately lead to the production of inflammatory cytokines (Szabo et al., 2007). Furthermore, TLR4 stimulation recruits cytoplasmic and membrane-bound adaptor proteins such as TIRAP and MyD88, facilitating activation of transcription factors (Kagan and Medzhitov, 2006). TIRAP is a key upstream inflammatory adaptor protein that participates in the majority of the TLR-mediated signalling events (Sakaguchi et al., 2011). It is known to engage in a variety of protein-protein interactions, playing a crucial role in the complex signalling events that underlie different inflammatory conditions (Srivastava et al., 2019). Owing to its critical role in TLR-mediated signalling pathways, TIRAP has emerged as a protein of interest in various inflammatory conditions. Although studies have implicated TIRAP involvement in the inflammatory conditions, its precise role in the pathogenesis of alcoholic liver fibrosis is not well understood. To further investigate the involvement of TIRAP in alcoholic liver fibrosis, we first assessed ethanol-mediated TIRAP activation in THP-1 macrophages, which are major inflammatory cells. Immunofluorescence analysis in THP-1 macrophages revealed significant activation of TIRAP upon exposure to ethanol, LPS, and LE (Figures 1A–C). These results provide evidence that ethanol can independently induce TIRAP activation in macrophages, establishing TIRAP involvement in alcoholic conditions and demonstrating TIRAP activation can be elicited as a primary response to ethanol exposure. Consistent with the role of TIRAP, Zhou et al. have suggested that myeloid-specific MyD88 deletion, the immediate downstream adaptor protein of TIRAP, shows a significant reduction in ethanol-induced liver injury in mice (Zhou et al., 2017). Furthermore, mice lacking TLR4 or MyD88 exhibited attenuated ethanol-induced neuroinflammation (Holloway et al., 2023). To further dissect ethanol-mediated TIRAP signalling, we analysed the effect of ethanol on MAPK activation. We observed that exposure of THP-1 macrophage to ethanol, LPS, and LE resulted in significant activation of all the major MAPKs, such as p38, JNK, and ERK (Figure 2A). This is consistent with previous studies, Zhang et al. suggested that LPS and ethanol both upregulate the phospho-p38 and phospho-ERK levels and activate NF-kB signalling pathway in alcoholic model (Zhang et al., 2024). Moreover, rat macrophages exposed to ethanol and LPS together show significant upregulation of p38 and JNK phosphorylation (Thakur et al., 2006). In TIRAP-driven macrophage inflammation, MAPKs activation is a key step, primarily responsible for the activation of transcription factors (Baig et al., 2017). We further investigated the activation of NF-κB in alcoholic conditions. Results demonstrated that ethanol, LPS, and LE induced significant phosphorylation of NF-kB (Figures 2A–C). Alcohol stimulates the production of reactive oxygen species, which can activate the NF-κB transcription factor in HCC (Wang et al., 2015). Consistent with this result, Mandrekar et al. have suggested that acute ethanol exposure to human monocytes can upregulate NF-kB activation (Mandrekar et al., 2007). To investigate the influence of ethanol on the mRNA levels of cytokines in THP-1 macrophages, we conducted RT-PCR analysis. It is interesting to note that we observed all the important cytokines, such as IL-β, IL-6 and TNF-α were upregulated in the presence of LPS, ethanol, and LE. Furthermore, results also demonstrated that the expression of growth factors such as TGF-β and PDGF-α was also significantly increased in response to all the conditions (Figure 2D). The data reported herein are also supported by previous findings, which demonstrated increased TGF-β levels in the blood of alcoholic patients, resulting in deleterious effects such as hepatic fibrosis and suppressed neuronal development (Kim et al., 2009). Additionally, experimental evidence demonstrates that recombinant TGF-β treatment in LX-2 cells elevates Smad2/3 and NOX4 expression alongside intracellular ROS accumulation, which subsequently triggers NLRP3 inflammasome activation to drive the HSC phenotype (Kang et al., 2022). Corroborating this mechanism, numerous pharmacological inhibition studies targeting the TGF-β pathway have successfully attenuated HSC activation, ultimately suppressing downstream liver fibrosis (Yang et al., 2021; Qin et al., 2024; Meng et al., 2025). Interestingly, our results indicate that alcohol-treated macrophages exhibit mixed activation phenotypes, characterized by concomitant upregulation of pro-inflammatory cytokines and profibrotic cytokines such as IL-β, IL-6 and TNF-α as TGF-β and PDGF-α. Another study similarly reported that PBMCs isolated from patients with alcoholic hepatitis exhibited elevated levels of both M1 and M2 markers. This phenomenon highlights the intricate interaction between ethanol and macrophages (Kim et al., 2019). Therefore, we determined how ethanol induces TIRAP-mediated THP-1 macrophage activation involved in the pathogenesis of liver fibrosis. To do so, we collected conditioned media from THP-1 macrophages exposed to LPS, ethanol, and LE. Then we treated the HSCs with the conditioned media generated from the mentioned conditions and checked the expression of HSC activation markers, α-SMA and collagen. Results demonstrate that HSCs treated with conditioned media derived from LPS, ethanol, LE showed significant morphological differences represented by an arrow in Figure 3A. Further HSC activation was confirmed by increased α-SMA and collagen expression in similar conditions (Figures 3B–F). These findings indicate that ethanol-induced TIRAP mediates THP-1 macrophage activation, stimulates HSC activation, and upregulates expression of extracellular matrix proteins, which are a prominent marker for liver fibrosis. These findings suggest that this robust HSC activation is a cumulative effect of multiple components within the conditioned media, including secreted proteins, cytokines, and exosomes, etc. A constraint of our current approach, however, is that a comprehensive biochemical characterization of the conditioned medium itself remains to be fully established. Subsequently, to support this macrophage-specific TIRAP-mediated indirect HSC activation under alcoholic conditions, we knocked down TIRAP in macrophages and assessed its effect on HSC activation. We found that CM derived from TIRAP-knockdown macrophages was unable to induce HSC activation. This was confirmed by the estimation of α-SMA and collagen protein expression (Figure 5). Since MyD88 lies downstream of TIRAP, its deletion showed similar results in alcoholic liver disease, which is well aligned with our study. Zhou et al. have suggested that myeloid-specific deletion of MyD88 in mice makes them resistant to developing ethanol-induced liver injury and prevents alcoholic hepatitis and hepatic inflammation (Zhou et al., 2017). However, while TIRAP’s role in generalized TLR inflammation is well-known, our study provides a novel mechanistic advance by demonstrating that macrophage TIRAP acts as the upstream regulator driving macrophage-HSC paracrine crosstalk in ALD. Crucially, we show that silencing macrophage TIRAP is sufficient to significantly attenuates this intercellular loop and reduces HSC activation. Ultimately, these findings transition TIRAP from a known inflammatory adapter into a preferential, novel therapeutic checkpoint to suppress the progression of alcoholic liver fibrosis.

## 5 Conclusion

Alcoholic liver fibrosis is a progressive condition characterized by the excessive accumulation of scar tissue in the liver, leading to impaired liver function. This study demonstrates that TIRAP-mediated signaling in macrophages plays a crucial role in the activation of hepatic stellate cells during alcoholic liver disease. Ethanol and LPS treatment of macrophages trigger inflammatory and fibrogenic responses by increasing phosphorylation of TIRAP and its downstream targets, including MAPK and NF-κB, which, in turn, promote HSC activation and fibrogenesis. Although the precise profile of secreted mediators in macrophage-conditioned medium remains to be fully characterized, the significant reduction in fibrotic markers following TIRAP silencing underscores its pivotal role in this cellular interplay. Consequently, targeting the TIRAP pathway represents a promising therapeutic strategy for antifibrotic treatments, warranting further investigation into the specific macrophage-derived soluble factors mediating this response.

## Data Availability

The original contributions presented in the study are included in the article/Supplementary Material, further inquiries can be directed to the corresponding author.

## References

[B1] Acharya P. Chouhan K. Weiskirchen S. Weiskirchen R. (2021). Cellular mechanisms of liver fibrosis. Front. Pharmacol. 12, 671640. 10.3389/fphar.2021.671640 34025430 PMC8134740

[B2] Atre R. Sharma R. Vadim G. Solanki K. Wadhonkar K. Singh N. (2023). The indispensability of macrophage adaptor proteins in chronic inflammatory diseases. Int. Immunopharmacol. 119, 110176. 10.1016/j.intimp.2023.110176 37104916

[B3] Baig M. S. Liu D. Muthu K. Roy A. Saqib U. Naim A. (2017). Heterotrimeric complex of p38 MAPK, PKCδ, and TIRAP is required for AP1 mediated inflammatory response. Int. Immunopharmacol. 48, 211–218. 10.1016/j.intimp.2017.04.028 28528205

[B4] Berumen J. Baglieri J. Kisseleva T. Mekeel K. (2021). Liver fibrosis: pathophysiology and clinical implications. WIREs Mech. Dis. 13, e1499. 10.1002/wsbm.1499 32713091 PMC9479486

[B5] Fang H. Pengal R. A. Cao X. Ganesan L. P. Wewers M. D. Marsh C. B. (2004). Lipopolysaccharide-induced macrophage inflammatory response is regulated by SHIP. J. Immunol. 173, 360–366. 10.4049/jimmunol.173.1.360 15210794

[B6] Ginès P. Krag A. Abraldes J. G. Solà E. Fabrellas N. Kamath P. S. (2021). Liver cirrhosis. Lancet 398, 1359–1376. 10.1016/s0140-6736(21)01374-x 34543610

[B7] Holloway K. N. Douglas J. C. Rafferty T. M. Kane C. J. M. Drew P. D. (2023). Ethanol induces neuroinflammation in a chronic Plus binge mouse model of alcohol use disorder via TLR4 and MyD88-Dependent signaling. Cells 12, 2109. 10.3390/cells12162109 37626919 PMC10453365

[B8] Ju C. Tacke F. (2016). Hepatic macrophages in homeostasis and liver diseases: from pathogenesis to novel therapeutic strategies. Cell. Mol. Immunol. 13, 316–327. 10.1038/cmi.2015.104 26908374 PMC4856798

[B9] Kagan J. C. Medzhitov R. (2006). Phosphoinositide-mediated adaptor recruitment controls toll-like receptor signaling. Cell 125, 943–955. 10.1016/j.cell.2006.03.047 16751103

[B10] Kang H. Seo E. Oh Y. S. Jun H.-S. (2022). TGF-β activates NLRP3 inflammasome by an autocrine production of TGF-β in LX-2 human hepatic stellate cells. Mol. Cell. Biochem. 477, 1329–1338. 10.1007/s11010-022-04369-5 35138513 PMC8989865

[B11] Kim Y.-K. Lee B. C. Ham B. J. Yang B.-H. Roh S. Choi J. (2009). Increased transforming growth Factor-beta1 in alcohol dependence. J. Korean Med. Sci. 24, 941–944. 10.3346/jkms.2009.24.5.941 19794996 PMC2752781

[B12] Kim A. Saikia P. Nagy L. E. (2019). miRNAs involved in M1/M2 hyperpolarization are clustered and coordinately expressed in alcoholic hepatitis. Front. Immunol. 10, 1295. 10.3389/fimmu.2019.01295 31231396 PMC6568035

[B13] Liu S. Feng G. Wang G. Liu G. (2008). p38MAPK inhibition attenuates LPS-Induced acute lung injury involvement of NF-κB pathway. Eur. J. Pharmacol. 584, 159–165. 10.1016/j.ejphar.2008.02.009 18328478

[B14] Mandrekar P. Jeliazkova V. Catalano D. Szabo G. (2007). Acute alcohol exposure exerts anti-inflammatory effects by inhibiting IκB kinase activity and p65 phosphorylation in human monocytes. J. Immunol. 178, 7686–7693. 10.4049/jimmunol.178.12.7686 17548605

[B15] Meng Z. Qiu X. Chen Z. Lee Y. Zhou L. Lu Y. (2025). Myeloid TGF-β signaling shapes liver macrophage heterogeneity and metabolic liver disease pathogenesis. JHEP Rep. 7, 101488. 10.1016/j.jhepr.2025.101488 40704067 PMC12284356

[B16] Park B. S. Lee J.-O. (2013). Recognition of lipopolysaccharide pattern by TLR4 complexes. Exp. Mol. Med. 45, e66. 10.1038/emm.2013.97 24310172 PMC3880462

[B17] Parlesak A. Schäfer C. Schütz T. Bode J. C. Bode C. (2000). Increased intestinal permeability to macromolecules and endotoxemia in patients with chronic alcohol abuse in different stages of alcohol-induced liver disease. J. Hepatol. 32, 742–747. 10.1016/S0168-8278(00)80242-1 10845660

[B18] Pastore M. Caligiuri A. Raggi C. Navari N. Piombanti B. Di Maira G. (2022). Macrophage MerTK promotes profibrogenic cross-talk with hepatic stellate cells via soluble mediators. JHEP Rep. 4, 100444. 10.1016/j.jhepr.2022.100444 35252828 PMC8891698

[B19] Poynard T. Mathurin P. Lai C.-L. Guyader D. Poupon R. Tainturier M.-H. (2003). A comparison of fibrosis progression in chronic liver diseases. J. Hepatol. 38, 257–265. 10.1016/S0168-8278(02)00413-0 12586290

[B20] Qin D. Han C. Gao Y. Li H. Zhu L. (2024). Lactucin reverses liver fibrosis by inhibiting TGF-β1/STAT3 signaling pathway and regulating short-chain fatty acids metabolism. Sci. Rep. 14, 19323. 10.1038/s41598-024-70253-5 39164375 PMC11336071

[B21] Rajpoot S. Wary K. K. Ibbott R. Liu D. Saqib U. Thurston T. L. M. (2021). TIRAP in the mechanism of inflammation. Front. Immunol. 12, 697588. 10.3389/fimmu.2021.697588 34305934 PMC8297548

[B22] Sakaguchi M. Murata H. Yamamoto K. Ono T. Sakaguchi Y. Motoyama A. (2011). TIRAP, an adaptor protein for TLR2/4, transduces a signal from RAGE phosphorylated upon ligand binding. PLoS ONE 6, e23132. 10.1371/journal.pone.0023132 21829704 PMC3148248

[B23] Srivastava M. Saqib U. Banerjee S. Wary K. Kizil B. Muthu K. (2019). Inhibition of the TIRAP-c-Jun interaction as a therapeutic strategy for AP1-mediated inflammatory responses. Int. Immunopharmacol. 71, 188–197. 10.1016/j.intimp.2019.03.031 30909134

[B24] Suraweera D. B. Weeratunga A. N. Hu R. W. Pandol S. J. Hu R. (2015). Alcoholic hepatitis: the pivotal role of kupffer cells. World J. Gastrointest. Pathophysiol. 6, 90–98. 10.4291/wjgp.v6.i4.90 26600966 PMC4644891

[B25] Szabo G. Dolganiuc A. Dai Q. Pruett S. B. (2007). TLR4, ethanol, and lipid rafts: a new mechanism of ethanol action with implications for other receptor-mediated effects. J. Immunol. 178, 1243–1249. 10.4049/jimmunol.178.3.1243 17237368

[B26] Tan Y. Wang Z. Guo R. Zhou X. Zhang W. Wu M. (2024). Dual-targeting macrophages and hepatic stellate cells by modified albumin nanoparticles for liver cirrhosis treatment. ACS Appl. Mat. Interfaces 16, 11239–11250. 10.1021/acsami.3c17670 38395769

[B27] Thakur V. Pritchard M. T. McMullen M. R. Wang Q. Nagy L. E. (2006). Chronic ethanol feeding increases activation of NADPH oxidase by lipopolysaccharide in rat kupffer cells: role of increased reactive oxygen in LPS-Stimulated ERK1/2 activation and TNF-α production. J. Leukoc. Biol. 79, 1348–1356. 10.1189/jlb.1005613 16554353 PMC1959405

[B28] Wang F. Yang J.-L. Yu K. Xu M. Xu Y. Chen L. (2015). Activation of the NF-κB pathway as a mechanism of alcohol enhanced progression and metastasis of human hepatocellular carcinoma. Mol. Cancer 14, 10. 10.1186/s12943-014-0274-0 25622857 PMC4320626

[B29] Widowati W. Sabrina A. H. N. Sutendi A. F. Zahiroh F. H. Nurjamil A. M. Wargasetia T. L. (2026). Immune system and hepatic stellate cells’ crosstalk in liver fibrosis: pathways and therapeutic potential. J. Immunol. Res. 2026, 2656395. 10.1155/jimr/2656395 41487942 PMC12759114

[B30] Yang J. H. Ku S. K. Cho I. J. Lee J. H. Na C.-S. Ki S. H. (2021). Neoagarooligosaccharide protects against hepatic fibrosis via inhibition of TGF-β/Smad signaling pathway. Int. J. Mol. Sci. 22, 2041. 10.3390/ijms22042041 33670808 PMC7922480

[B31] Zhang C.-Y. Yuan W.-G. He P. Lei J.-H. Wang C.-X. (2016). Liver fibrosis and hepatic stellate cells: etiology, pathological hallmarks and therapeutic targets. World J. Gastroenterol. 22, 10512–10522. 10.3748/wjg.v22.i48.10512 28082803 PMC5192262

[B32] Zhang J. Liu Y. Chen H. Yuan Q. Wang J. Niu M. (2022). MyD88 in hepatic stellate cells enhances liver fibrosis via promoting macrophage M1 polarization. Cell Death Dis. 13, 411. 10.1038/s41419-022-04802-z 35484116 PMC9051099

[B33] Zhang Z. Leng Z. Kang L. Yan X. Shi J. Ji Y. (2024). Alcohol inducing macrophage M2b polarization in colitis by modulating the TRPV1-MAPK/NF-κB pathways. Phytomedicine 130, 155580. 10.1016/j.phymed.2024.155580 38810558

[B34] Zhou H. Yu M. Roychowdhury S. Sanz‐Garcia C. Pollard K. A. McMullen M. R. (2017). Myeloid‐MyD88 contributes to ethanol‐induced liver injury in mice linking hepatocellular death to inflammation. Alcohol. Clin. Exp. Res. 41, 719–726. 10.1111/acer.13345 28165624 PMC5391031

