# Supplementary material for: Toll/IL-1 receptor domain-containing adaptor protein plays a crucial role in macrophage-mediated hepatic stellate cell activation in alcoholic liver disease
Source: Front Cell Dev Biol. 2026 Aug 4;14:1883301. doi: 10.3389/fcell.2026.1883301 (PMC13481357; doi:10.3389/fcell.2026.1883301)
Supplement: Supplementary file 1 [file DataSheet1.docx]

**Supplementary data**

**Alcoholic Murine model**

Animals were housed in ventilated cages under controlled 12 hrs light/dark cycle with access of standard mice food and water. This study was performed according to the animal protocol which was approved by the Institutional Animal Ethics Committee (IAEC) of Acropolis Institute of Pharmaceutical Education and Research (AIPER) and following the policies of the Committee for the Purpose of Control and Supervision of Experiments on Animals (CPCSEA), Government of India. To generate alcoholic mice model, the animals were given ad libitum (free) access to a 25% (v/v) ethanol solution as their primary source of drinking water for 3 months. No forced administration, gavage, or aversion techniques were utilized, ensuring that the ethanol intake remained entirely voluntary. Further to confirm the development of alcoholic model all the serological test and H&E staining was done.

**Results**

**Development of alcoholic liver fibrosis mice model**

To development of chronic alcoholic liver fibrosis, C57BL/6 mice were administered 25% ethanol in drinking water for 3 months. Serum was isolated after the development of alcoholic liver fibrosis. First, we investigate the pathology associated with ethanol feeding, liver and other tissue sample were harvested from both groups. H&E staining of liver tissue indicated well organized tissue morphology and liver architecture. In contrast, ethanol-treated mice exhibited marked liver damage evidenced by hepatocellular ballooning and disruption on normal liver architecture (Fig. S1A). To further investigate the pathology associated with ethanol feeding, we analysed serum markers of liver and systemic injury, including ALT, AST. Serological profiling revealed a significant increase in ALT and AST in the ethanol-treated mice compared to the control group suggested significant liver damage and hepatic inflammation (Fig. S1B). Next, we examined TIRAP activation and its downstream transcription factor, NF-kB, to elucidate the molecular mechanisms in alcoholic mice. Western blotting demonstrated enhanced phosphorylation and activation of both proteins (Fig.S1C). Furthermore, to confirm the fibrosis progression we analysed the expression of fibrotic markers such as α-SMA and collagen. Liver tissue immunohistochemistry revealed significant elevation in both the fibrotic markers (Fig. S1E-F). Generally, these data indicate the TIRAP mediated signaling plays a crucial role in the development of alcoholic liver fibrosis mice model.

**Figure S1.** Ethanol consumption induces liver injury, activates TIRAP/NF-kB signaling, and promotes fibrotic markers in mice. **(A)** Representative haematoxylin and eosin (H&E) staining of liver sections from control and ethanol-treated mice at 40X magnification. The ethanol group exhibits prominent histological alterations, including macrovascular steatosis and cellular infiltration.**(B)** Serum levels of aspartate aminotransferase (AST) and alanine aminotransferase (ALT) expressed as fold change relative to the control group (n= 3 per group).**(C)** Western blot analysis of p-TIRAP and p-P65 in liver tissue and quantification via image j software (D) Representative immunofluorescence images of liver sections stained for α-smooth muscle actin (α -SMA, green) and counterstained with DAPI (blue) for nuclear visualization under bright-field, fluorescence, and merged channels.(E) Representative immunofluorescence images demonstrating collagen deposition (red) and DAPI (blue) in the liver tissue of control and alcoholic mice across bright-field, fluorescence, and merged fields. Data are presented as mean. *P < 0.05, * *P < 0.01 compared with the control group.

**Figure S2.** HSCs Morphology of HSCs treated with macrophage condition media derived from wild type and TIRAP-knocked out THP-1 macrophages exposed to LPS, ethanol and LE for 24hrs. HSCs treated with THP-1 macrophage-conditioned media exhibit distinct morphological changes compared to controls, while HSCs exposed to LPS, ethanol, and LE-conditioned media display prominent star-shaped and spiky morphology, as highlighted by the red arrow.

**Table 1.- List of primers.**

| **Sr. No.** | **Primer** | **Sequence** | **Accession Numbers** |
| --- | --- | --- | --- |
| **1.** | IL-1β F | ACCACTACAGCAAGGGCTTC | BT007213.1 |
| **2.** | IL-1β R | TCGTCGACATAAGCCTCGTT |  |
| **3.** | IL-6 F | TTCAATGAGGAGACTTGCCTGG | M54894.1 |
| **4.** | IL-6 R | CTGGCATTTGTGGTTGGGTC |  |
| **5.** | TNF-α F | CCCATGTTGAGCAAACCCTC | NM_000594.4 |
| **6.** | TNF-α R | TGAGGTACAGGCCCTCTGAT |  |
| **7.** | TGF-β F | ACAGCAAACAATTCCTGGCGA | NM_000660.7 |
| **8.** | TGF-β R | CAATTTCCCCTCCACGGCTC |  |
| **9.** | PDGF- α F | CACACCTCCTCGCTGTAGTATTTA | NG_029727.1 |
| **10.** | PDGF-α R | GTTATCGGTGTAAATGTCATCCAA |  |
| **11.** | α-SMA F | CCTATCCCCGGGACTAAGAC | NM_001406471.1 |
| **12.** | α-SMA R | AGGCAGTGCTGTCCTCTTCT |  |
| **13.** | Collagen 1A1 F | CGCTGAGCCTGTTTCCCTTT | D21337.1 |
| **14.** | Collagen 1A1 R | ACAACGTAACCAGGAGCAGC |  |
| **15.** | GAPDH F | AATCCCATCACCATCTTCCA | NC_000012.12 |
| **16.** | GAPDH R | TGGACTCCACGACGTACTCA |  |
| **17.** | TIRAP F | GCGCAGGCCTTACATAGGAA | AK124298.1 |
| **18.** | TIRAP R | AGACCAGGCATTGGTGAAGG |  |

**Table 2.- List of primary and secondary antibodies.**

| **Sr. No.** | **Antibodies** | **Catalogue Number and Make** |
| --- | --- | --- |
| **1.** | TIRAP | SC-166149 Santa Cruze |
| **2.** | Phospho-TIRAP | Bs-7562R |
| **3.** | NF-kB | SC-8008 Santa Cruze |
| **4.** | Phospho-NF-kB | 3033S Cell Signalling |
| **5.** | P38 | 9212S Cell Signalling |
| **6.** | Phospho-p38 | SC-17852-R |
| **7.** | ERK | 9102S Cell Signalling |
| **8.** | Phospho-ERK | 9101S Cell Signalling |
| **9.** | JNK | 9252S Cell Signalling |
| **10.** | Phospho-JNK | 9251S Cell Signalling |
| **11.** | Smooth Muscle Actin | SC-53015 Santa Cruze |
| **12.** | Collagen 1A1 | SC-8784 Santa Cruze |
| **13.** | β-Actin | SC-47778 Santa Cruze |
